# Heterointerface Seeding Accelerates Bi^0^/Bi^3+^ Phase Conversion for Ultrafast Aqueous Alkaline Batteries

**DOI:** 10.1002/advs.78066

**Published:** 2026-09-30

**Authors:** Jingwen Ma, Teng Wang, Jiaye Ye, Tong Yang, Zhongguo Zhao, Yuanyou Peng, Meimei Yu, Chaojiang Fan, Wenhu Li, Hongxia Wang

**Affiliations:** ^1^ School of Materials Science and Engineering Shaanxi University of Technology Hanzhong Shaanxi China; ^2^ School of Chemistry and Physics Faculty of Science Queensland University of Technology Brisbane Queensland Australia

**Keywords:** aqueous alkaline batteries, Bi/Bi_2_O_3_ heterojunction, fast charging, pre‐seeded nuclei

## Abstract

Ultrafast aqueous alkaline batteries (AABs) require anodes capable of sustaining rapid multielectron redox kinetics under extreme current densities. Although bismuth oxides offer high theoretical capacities, their high‐rate performance is constrained by the large nucleation barrier during phase transitions. This kinetic barrier remains unresolved by conventional charge‐transport engineering. Herein, one‐dimensional heterointerface‐seeded Bi/Bi_2_O_3_@C nanowires are developed to regulate the phase‐transition pathway. Ex situ/operando characterizations and theoretical analyses reveal that persistently retained Bi/Bi_2_O_3_ heterointerfaces serve as pre‐existing nucleation sites, enabling bidirectional Bi^0^/Bi^3+^ conversion via interfacial regrowth rather than repeated homogeneous nucleation. This seeded conversion pathway substantially reduces phase‐transition polarization, while the nitrogen‐doped carbon shell provides continuous electron/OH^−^ transport pathways and suppresses structural degradation. Consequently, the Bi/Bi_2_O_3_@C anode delivers a specific capacity of 295.7 mAh g^−1^ at 1 A g^−1^ and maintains 123.1 mAh g^−1^ even at 150 A g^−1^, alongside a capacity retention of 82.4% after 1000 cycles at 100 A g^−1^. This robust rate capability is preserved even at a high mass loading of 12.4 mg cm^−2^. Full AAB cells paired with a CoOOH/Co(OH)_2_/CoO cathode further demonstrate the device‐level feasibility of the proposed architecture. This heterointerface‐seeding strategy offers a general design principle for high‐capacity electrode materials operating under ultrafast‐charging conditions.

## Graphical abstract

An N‐doped carbon‐confined Bi/Bi_2_O_3_ heterostructure enables ultrafast and reversible Bi^0^/Bi^3+^ phase conversion through heterointerface seeding. The pre‐established Bi/Bi_2_O_3_ interfaces provide nucleation‐active sites for heterophase regrowth, while the N‐C shell promotes electron transport and structural stability, overcoming kinetic limitations in aqueous alkaline batteries.

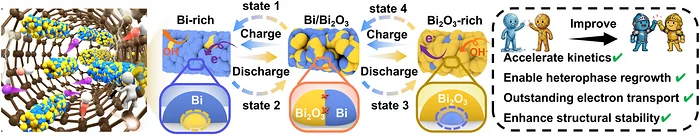

## 1 Introduction

Aqueous alkaline batteries (AABs) are promising for large‐scale and high‐power energy storage owing to their intrinsic safety, low cost, and rapid ion‐transport kinetics [1, 2, 3]. Although AAB cathodes have been extensively investigated [4, 5, 6], the development of compatible anodes capable with ultrafast conversion kinetics remains a critical bottleneck. Conventional anodes (e.g., Zn and Fe) inherently suffer from parasitic hydrogen evolution, surface corrosion, and dendrite growth under high‐current regimes [7, 8, 9, 10, 11]. Therefore, developing anode materials that couple high capacity and fast reaction kinetics with stable structural evolution is essential to advance high‐power AABs [12, 13].

Bismuth (Bi)‐based conversion materials are emerging as highly promising anode candidates owing to their relatively low redox potential, high theoretical capacity, and multi‐electron redox activity. In particular, Bi_2_O_3_ undergoes reversible Bi^0^/Bi^3+^ conversion within a favorable potential window of approximately −1.0 to 0 V vs. Hg/HgO, delivering a theoretical capacity of 345.1 mAh g^−1^ [14, 15, 16]. However, under demanding high‐rate charge/discharge conditions, pristine Bi_2_O_3_ suffers from severe capacity decay owing to its low intrinsic electronic conductivity, pronounced phase‐transition polarization, and structural degradation upon repeated cycles [17, 18]. To overcome these limitations, various materials‐engineering strategies, including metal‐organic framework (MOF)‐derived porous architectures [19, 20, 21, 22], oxygen‐vacancy engineering [23] and interfacial engineering [24, 25, 26, 27, 28, 29], have been developed to enhance electron/ion transport and buffer volume variations. For instance, Wang et al. [18] demonstrated that constructing a 0D/2D amorphous/crystalline BiO_x_‐Bi heterostructure effectively mitigate high‐rate capacity decay, retaining 79.9% capacity upon a 20‐fold increase in current density. While these strategies can improve apparent reaction kinetics, they do not fundamentally address the intrinsic phase‐transition barriers that limit high‐rate Bi^0^/Bi^3+^ conversion.

Fundamentally, the reaction kinetics of conversion‐type electrodes are not governed solely by bulk electron/ion transport. Because these reactions involve profound structural reconstruction, the kinetic barriers for crystal nucleation and the subsequent growth of new phases primarily dictate phase‐transition polarization and high‐rate kinetics. Such nucleation‐governed behaviors are well established in Li, Na, and Zn metal anodes, where lowering nucleation barriers or introducing heterogeneous seeding sites significantly enhances high‐rate reversibility [30, 31, 32, 33]. Inspired by this concept, the reversible Bi/Bi_2_O_3_ conversion, which inherently involves repeated phase formation and growth during deep redox reactions, may likewise be influenced by nucleation‐associated phase‐transition barriers during Bi/Bi_2_O_3_ conversion. Nevertheless, the precise roles of nucleation and heterophase interfacial regrowth in governing high‐rate Bi/Bi_2_O_3_ conversion kinetics remain insufficiently explored. Therefore, we propose that, under high‐current regimes, the sluggish bidirectional Bi/Bi_2_O_3_ conversion is strongly constrained by the energy‐demanding homogeneous nucleation step, beyond conventional bulk charge‐transport limitations.

Herein, to overcome the aforementioned nucleation‐dominated kinetic limitations, Bi/Bi_2_O_3_ heterogeneous nanowires are constructed to enable a reversible heterojunction interfacial conversion mechanism. During deep redox cycles, the retained heterogeneous interfaces serve as pre‐existing nucleation sites, guiding the bidirectional Bi^0^/Bi^3+^ conversion via interfacial regrowth, thereby reducing the reliance on homogeneous nucleation. This pathway effectively reduces phase‐transition barriers and accelerates high‐rate reaction kinetics. Furthermore, the system is integrated with an N‐doped carbon coating (Bi/Bi_2_O_3_@C) to establish continuous electron/OH^−^ transport networks and structural confinement, preserving kinetic advantages under high current density and high mass loading conditions. Consequently, the optimized Bi/Bi_2_O_3_@C electrode delivers a specific capacity of 295.7 mAh g^−1^ at 1 A g^−1^ and retains 123.1 mAh g^−1^ even at 150 A g^−1^. Notably, this high‐rate performance outperforms most state‐of‐the‐art Bi‐based anodes. High‐mass loading experiments further demonstrate the robust rate capability of Bi/Bi_2_O_3_@C samples, achieving a capacity retention of 96.2% from 1 to 25 A g^−1^ even at a mass loading of 12.4 mg cm^−2^. The practical viability of this electrode design is validated through the assembly of AAB full batteries and pouch cells. This pre‐seeded heterojunction strategy provides a rational pathway for the interfacial design of high‐rate conversion‐type electrodes.

## 2 Results and Discussion

### 2.1 Synthesis and Structural Characterization

As illustrated in Figure 1a, the one‐dimensional Bi/Bi_2_O_3_ heterojunction (Bi/Bi_2_O_3_ HJ) and Bi/Bi_2_O_3_@C nanowires are fabricated through a template‐directed topotactic strategy. Initially, a Bi‐MOF nanowire precursors is synthesized through a solvothermal method, serving as both the structural scaffold and a uniformly distributed Bi source [34]. Subsequent calcination induces a topological conversion; the reducing atmosphere, generated from the decomposition of the organic ligands, partially reduces Bi^3+^ to metallic Bi^0^, while the remaining Bi^3+^ converts into Bi_2_O_3_, thereby yielding in situ‐formed Bi/Bi_2_O_3_ heterostructure nanowires [21]. In this architectural design, the abundant heterointerfaces are expected to act as pre‐existing nucleation sites. During electrochemical cycling, these interfaces can guide the bidirectional Bi^0^/Bi^3+^ conversion via direct interfacial regrowth, strategically bypassing the energy‐intensive homogeneous nucleation step. Finally, a nitrogen‐doped carbon (N—C) layer is constructed on the surface of Bi/Bi_2_O_3_ through polydopamine (PDA) coating and subsequent carbonization. This process yields carbon‐confined Bi/Bi_2_O_3_@C nanowires, an architecture engineered to establish continuous electron transport networks and buffer structural volume variations during high‐rate conversion [35].

**FIGURE 1:**
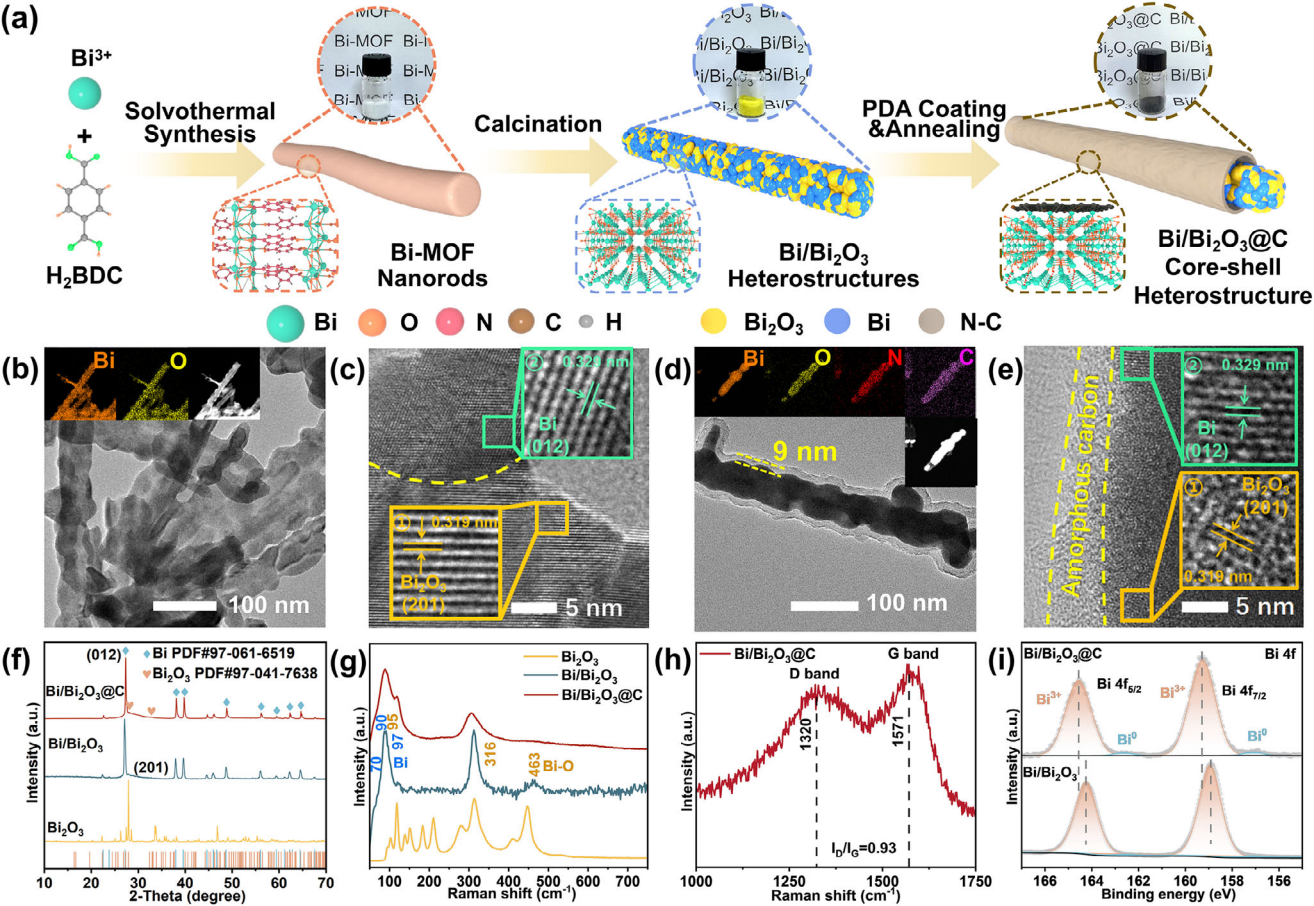
(a) Schematic illustration of the preparation of Bi/Bi_2_O_3_@C. (b) TEM and EDS mapping and (c) HRTEM image of Bi/Bi_2_O_3_. (d) TEM and EDS mapping and (e) HRTEM image of Bi/Bi_2_O_3_@C. (f) XRD patterns, (g,h) Raman spectra. (i) Bi 4f XPS spectra of Bi/Bi_2_O_3_ and Bi/Bi_2_O_3_@C.

Scanning electron microscopy (SEM) and transmission electron microscopy (TEM) were performed to characterize the morphological and microstructural features of the pre‐seeded Bi/Bi_2_O_3_@C nanowires and control samples. The Bi‐MOF precursor exhibits a uniform nanowire morphology with a high aspect ratio (Figure S1a), providing a structural template for the subsequent formation of the Bi/Bi_2_O_3_ heterostructure. Upon thermal treatment, the resulting Bi/Bi_2_O_3_ nanowires largely inherits the one‐dimensional morphology of the Bi‐MOF precursor (Figure 1b and Figure S1b). The corresponding EDS elemental mapping reshows that Bi and O are distributed throughout the nanowire with substantial spatial overlap, indicating that the Bi and Bi_2_O_3_ are integrated within the same one‐dimensional framework (Figure 1b). Furthermore, high‐resolution TEM (HRTEM) resolves distinct lattice fringes with spacings of 0.329 and 0.319 nm, corresponding to the (012) plane of metallic Bi and the (201) plane of Bi_2_O_3_, respectively (Figure 1c). More detailed HRTEM analysis of multiple regions identifies the Bi (012), (110), and (104) planes and Bi_2_O_3_ (201) and (220) planes, respectively, revealing that Bi and Bi_2_O_3_ domains are closely intergrown and in intimate contact within the nanowire (Figure S2). This crystallographic evidence confirms the in‐situ formation of abundant Bi/Bi_2_O_3_ heterointerfaces within the nanowires. These interfacial regions are structurally poised to provide nucleation and interfacial regrowth sites for electrochemical phase transitions, laying the physical foundation for accelerated high‐rate kinetics [21, 22]. Following the polydopamine (PDA) coating and subsequent carbonization, TEM characterization of the resulting Bi/Bi_2_O_3_@C nanowires reveals a continuous amorphous coating layer with a thickness of approximately 9 nm surrounding the nanowire (Figure 1d bottom line). EDS mapping results reveal the homogeneous distribution of Bi and O within the nanowire core, while C and N are predominantly in the outer region, further supporting the formation of a core–shell architecture. The HRTEM image further distinguishes the amorphous carbon shell from the crystalline inner core. Distinct lattice fringes with spacings of the Bi (012) and Bi_2_O_3_ (201) planes, respectively, demonstrating that the PDA‐derived N‐C layer does not destroy the internal Bi/Bi_2_O_3_ heterojunction structure, but instead forms a protective and conductive shell around the heterojunction‐seeded nanowire core (Figure 1e). In contrast, the control Bi_2_O_3_ sample exhibits an agglomerated bulk morphology without the nanowire framework or heterointerfaces (Figure S1d).

X‐ray diffraction (XRD), Raman spectra and X‐ray photoelectron spectroscopy (XPS) were performed to elucidate the phase composition and surface chemical characteristics of the samples. As shown in Figure 1f, the main diffraction peaks of both Bi/Bi_2_O_3_ and Bi/Bi_2_O_3_@C samples can be assigned to crystalline Bi (PDF#97‐061‐6519) and Bi_2_O_3_ (PDF#97‐041‐7638) phases [36]. Raman spectra further corroborate the coexistence of Bi and Bi_2_O_3_ in both materials, revealing distinct Bi—O vibrational modes and Bi‐related peaks at approximately 70–97 cm^−1^ (Figure 1g) [25]. Furthermore, the disordered nature of the N—C shell in Bi/Bi_2_O_3_@C is evidenced by I_D_/I_G_ intensity ratio of 0.93, a structural feature that could facilitates OH^−^ adsorption (Figure 1h) [37]. The high‐resolution Bi 4f XPS spectra of Bi/Bi_2_O_3_ and Bi/Bi_2_O_3_@C exhibit two pairs of characteristic peaks corresponding to Bi^3+^ and B^0^ species (Figure 1i) [38]. The relatively weak intensity of Bi° compared to Bi^3+^ signals is attributed to the surface‐sensitive nature of XPS (with a limited probing depth of < 10 nm) and the inevitable surface oxidation of metallic Bi [39, 40].

Moreover, the XPS survey, along with the high‐resolution O 1s, C 1s, and N 1s spectra, confirms the successful introduction of the N‐doped carbon coating in Bi/Bi_2_O_3_@C sample (Figure S3). The abundant N/O‐containing functional groups within this layer can serve as polar sites to promote OH^−^ adsorption and transport, thereby facilitating high‐rate interfacial reaction kinetics [41]. Above structural and compositional characterizations verify the successful construction of Bi/Bi_2_O_3_ heterostructures and the N—C confinement layer in the final Bi/Bi_2_O_3_@C product, laying the physical foundation for the proposed interfacial conversion mechanism.

### 2.2 Electrochemical Contribution of Bi/Bi_2_O_3_ Heterojunction

To elucidate the electrochemical contribution of pre‐seeded Bi/Bi_2_O_3_ heterojunctions, comparative measurements of pristine Bi_2_O_3_ and Bi/Bi_2_O_3_ were conducted in a three‐electrode system. The galvanostatic charge–discharge (GCD) curves of Bi/Bi_2_O_3_ show distinct redox plateaus corresponding to multi‐step conversion reactions (Figure 2a) [27]. In the three‐electrode configuration, the cathodic and anodic branches correspond to the reduction and oxidation processes of Bi species, respectively, involving the reversible cleavage and reconstruction of Bi−O bonds accompanied by Bi^0^/Bi^3+^ valence conversion, rather than capacitive surface adsorption/desorption [18, 42]. As displayed in Figure 2b,c and Figure S4, Bi/Bi_2_O_3_ sample maintains well‐defined charge–discharge plateaus at current densities ranging from 1 to 70 A g^−1^, exhibiting remarkably higher electrochemical reversibility than pristine Bi_2_O_3_. The extended plateau around −0.5 V arises from the coupled Bi^3+^/Bi^2+^/Bi^0^conversion, with the metastable Bi_2_O_2_ intermediate being continuously formed and consumed [22]. Notably, an potential dip associated with nucleation overpotential can be observed during the low‐current reduction process (Figure 2c). Compared with pristine Bi_2_O_3_, the Bi/Bi_2_O_3_ electrode exhibits a substantially reduced nucleation overpotential, decreasing from 0.1079 to 0.0726 V (approximately 32.7% reduction). This phenomenon originates from the pre‐established Bi/Bi_2_O_3_ heterointerfaces, where residual Bi and Bi_2_O_3_ domains can serve as heterophase nucleation and regrowth sites during reversible Bi^0^/Bi^3+^ conversion. Consequently, the phase transformation preferentially proceeds through interfacial reconstruction rather than repeated homogeneous nucleation, thereby reducing the energy barrier for phase transition and accelerating redox kinetics.

**FIGURE 2:**
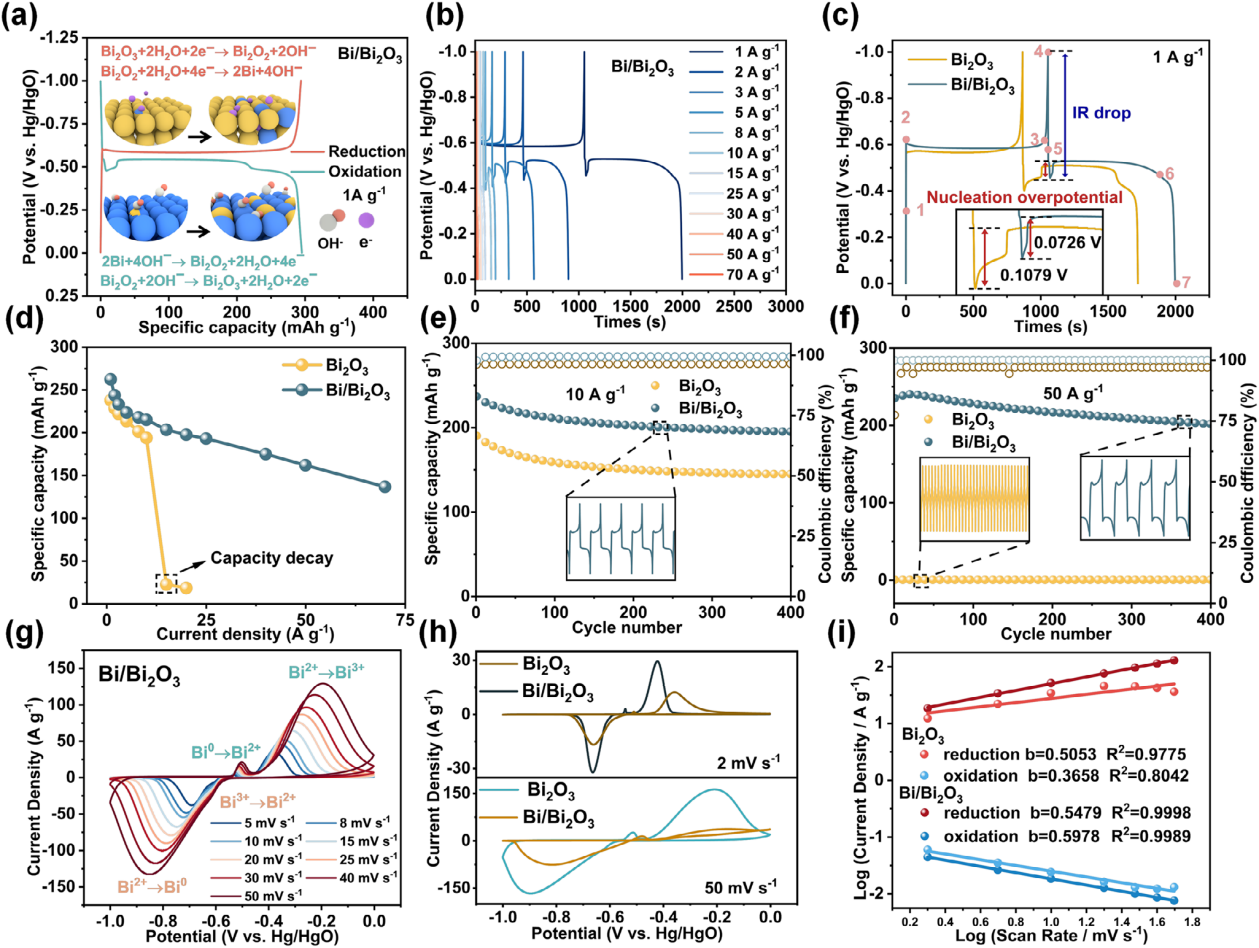
(a) Deep redox reaction mechanism of Bi/Bi_2_O_3_ at 1 A g^−1^. (b) GCD curves of Bi/Bi_2_O_3_ at 1–70 A g^−1^. (c) GCD curves of Bi_2_O_3_ and Bi/Bi_2_O_3_ at 1 A g^−1^. (d) Specific capacity of Bi_2_O_3_ and Bi/Bi_2_O_3_ at different current densities. (e) Cycling performance at 10 A g^−1^. (f) Cycling performance at 50 A g^−1^. (g) CV curves of Bi/Bi_2_O_3_ at 2–50 mV s^−1^. (h) CV curves of Bi_2_O_3_ and Bi/Bi_2_O_3_ at 2 and 50 mV s^−1^. (i) The relationship between the peak current and scan rates.

The corresponding rate performance calculated from GCD data (Figure S5) is illustrated in Figure 2d. At low current densities of 1–10 A g^−1^, both electrodes deliver comparable specific capacities, reaching 237.8 mAh g^−1^ and 262.3 mAh g^−1^ for pristine Bi_2_O_3_ and Bi/Bi_2_O_3_ at 1 A g^−1^, respectively. However, when the current density rises to 15 A g^−1^, the specific capacity of pristine Bi_2_O_3_ decreases sharply to 22.2 mAh g^−1^, whereas Bi/ Bi_2_O_3_ still retains 203.3 mAh g^−1^ (Figure 2d). Such a abrupt capacity decay of pristine Bi_2_O_3_ under ultrahigh current densities is mainly attributed to the sluggish nucleation and reconstruction process during the Bi^0^/Bi^3+^ conversion reaction. In the absence of pre‐existing Bi nuclei, pristine Bi_2_O_3_ undergoes a larger nucleation overpotential at 10 A g^−1^(0.1473 V vs. 0.0813 V for Bi/Bi_2_O_3_), leading to severe phase‐transition polarization and incomplete phase conversion under ultrafast discharge conditions (Figure S6a). Consequently, the discharge process of pristine Bi_2_O_3_ becomes markedly shortened at 10 A g^−1^ and almost disappears at 15 A g^−1^. In contrast, the pre‐established Bi/Bi_2_O_3_ heterointerfaces provide abundant nucleation‐active sites for Bi reconstruction, effectively lowering the nucleation barrier and facilitating reversible phase evolution (Figure S6b). Based on this, Bi/Bi_2_O_3_ delivers 136.5 mAh g^−1^ even at 70 A g^−1^, demonstrating the importance of heterointerface‐mediated nucleation regulation for high‐rate conversion reactions.

Cycling stability tests (Figure 2e,f) indicate that Bi/Bi_2_O_3_ delivers capacity retention of 82.1% and 85.8% after 400 cycles at 10 and 50 A g^−1^, respectively. In contrast, pristine Bi_2_O_3_ undergoes severe capacity decay under identical high current conditions (which dropped to 0 at 50 A g^−1^). Cyclic voltammetry (CV) profiles at scan rates of 2 – 50 mV s^−1^ (Figure 2g) show that Bi/Bi_2_O_3_ exhibits reversible redox behavior [43]. Specifically, taking the CV profile recorded at 5 mV s^−1^ as an example, the cathodic peaks centered at approximately −0.60 and −0.70 V are associated with the sequential reduction of Bi_2_O_3_ through possible low‐valence Bi intermediates toward metallic Bi. During the reverse oxidation process, the anodic peaks located at approximately −0.55 and −0.40 V are assigned to the oxidation of metallic Bi to low‐valence Bi species and the subsequent oxidation of these species to Bi_2_O_3_, respectively.

Notably, the lower‐potential redox pair is relatively weak, which may be attributed to the transient nature and limited accumulation of the low valence Bi species during the electrochemical conversion process [18, 20, 44]. It should be noted that the exact structural identity of these possible species requires more in‐depth investigation [17]. The two pairs of well‐defined redox peaks in the CV of Bi/Bi_2_O_3_ were respectively attributed to the following redox reactions [27].

The reduction reactions during this process are as following Equations (1) and (2):

(1) $$Bi_{2}O_{3}+H_{2}O+2e^{-}\;\to Bi_{2}O_{2}+2OH^{-}$$

(2) $$Bi_{2}O_{2}+2H_{2}O+4e^{-}\;\to \;2\mathrm{Bi}+4OH^{-}$$

The oxidation reactions during this process follows the Equations (3) and (4) below:

(3) $$2\mathrm{Bi}+4OH^{-}\;\to \;Bi_{2}O_{2}+2H_{2}O+4e^{-}$$

(4) $$Bi_{2}O_{2}+2OH^{-}\;\to \;Bi_{2}O_{3}+H_{2}O\;+2e^{-}$$

The overall reversible reaction can be described as Equation (5):

(5) $$Bi_{2}O_{3}+3H_{2}O+6e^{-}\;↔\;2\mathrm{Bi}+6OH^{-}$$

However, pristine Bi_2_O_3_ exhibits a markedly increased redox peak separation at elevated scan rates, indicating aggravated electrochemical polarization (Figure 2h and Figure S7). The charge storage mechanism of the Bi/Bi_2_O_3_ electrode was studied by CV testing at different scanning rates in the range of 2–50 mV s^−1^, with the *b‐*value calculated by using Equations (6) and (7) as follows:

(6) $$i=av^{b}\;$$

(7) log(i)=blog(v)+loga

where *i* is the peak current, *v* is the scan rate, and *a* and *b* are constants. The fitted *b*‐values fall between 0.5 and 1, indicating a mixed diffusion‐controlled and capacitive charge‐storage behavior [26]. Notably, Bi/Bi_2_O_3_ exhibits a higher *b*‐value for the oxidation peak than pristine Bi_2_O_3_ (0.60 vs. 0.37), indicating improved oxidation kinetics with reduced diffusion limitation (Figure 2i). Furthermore, quantitative charge storage analysis reveals a higher capacitive contribution (57.4% for Bi/Bi_2_O_3_ vs. 44.6% for Bi_2_O_3_ at 20 mV s^−1^, Figure S8). Together, these results indicate accelerated phase transition kinetics compared to the pristine counterpart [24]. Moreover, a comparison of the GCD curves at 1 A g^−1^ (Figure 2c, Figure S9) presents lower apparent nucleation overpotential and a smaller voltage (IR) drop for the Bi/Bi_2_O_3_. This lower overpotential suggests that the pre‐existing Bi/Bi_2_O_3_ interfaces may lower the nucleation barrier for Bi formation during the conversion reaction, thereby accelerating the reversible Bi^0^/Bi^3+^ conversion.

### 2.3 Rapid Conversion Mechanism of Bi/Bi_2_O_3_ Heterojunction Interfaces

To elucidate the phase evolution of the Bi/Bi_2_O_3_ electrode during the Bi^0^/Bi^3+^ conversion, *ex situ* XRD, XPS, HRTEM and Raman analyses were performed. The corresponding electrochemical states for *ex situ* characterizations were selected based on the GCD profile (Figure 2c). The *ex situ* XRD patterns reveal that the characteristic diffraction peaks of both Bi and Bi_2_O_3_ are retained throughout the entire charge/discharge process, accompanied by continuous variations in their relative intensities (Figure 3a) [26]. Rietveld refinement of the XRD data further quantifies this evolution of the Bi/Bi_2_O_3_ phase ratio: the respective relative fractions of Bi_2_O_3_ and Bi which are 70.40% and 29.60% at the open‐circuit state (state 1) change to 57.22% and 42.78% upon deep reduction (state 4), and then recover to 68.90% and 31.10% after subsequent deep oxidation (state 7) closely mirroring the initial composition (Figure 3b and Figure S10). *Ex situ* HRTEM characterization provides direct microscopic evidence for the persistent presence of the Bi/Bi_2_O_3_ heterophase interfaces throughout the conversion process (Figure S11). Metallic Bi and Bi_2_O_3_ lattice domains coexist at the deeply reduced state (state 4), a plateau state during subsequent oxidation (state 6), and the deeply oxidized state (state 7), despite the state‐dependent evolution of their relative distribution.

**FIGURE 3:**
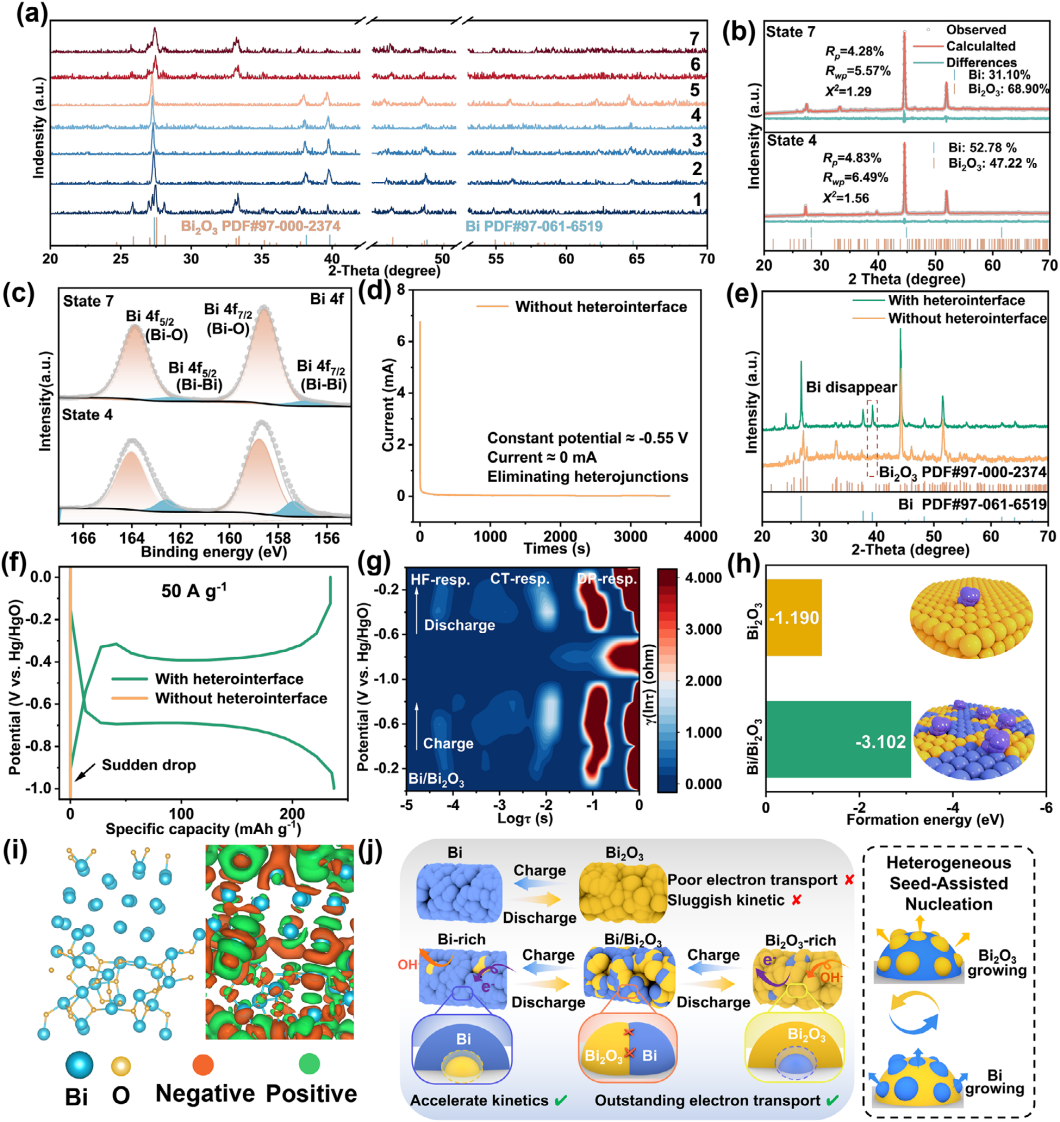
Structural characterization of Bi_2_O_3_ and Bi/Bi_2_O_3_ electrodes at various states at different charge/discharge states. (a) The *ex situ* XRD patterns of Bi/Bi_2_O_3_. (b) The *ex situ* XRD patterns and corresponding Rietveld refinement results of states 4 and 7. (c) *Ex situ* Bi 4f XPS spectra of Bi/Bi_2_O_3_ at states 4 and 7. (d) Chronoamperometric curve recorded at −0.55 V vs. Hg/HgO during electrochemical elimination of the Bi/Bi_2_O_3_ heterointerfaces. (e) XRD patterns before and after heterointerface elimination. (f) GCD curves at 50 A g^−1^ before and after heterointerface elimination. (g) *Operando* EIS‐derived DRT analysis of Bi/Bi_2_O_3_. (h) Formation energies for Bi growth on Bi_2_O_3_ surface and at the Bi/Bi_2_O_3_ heterointerface. (i) Charge density difference of the Bi/Bi_2_O_3_ heterointerface. (j) Schematic illustration of the proposed seed‐assisted heterogeneous nucleation at Bi/Bi_2_O_3_ heterointerfaces.

Furthermore, the persistent Bi−Bi and Bi−O vibrational signals in the *ex situ* Raman spectra (Figure S12), together with the concurrent presence of Bi^0^ and Bi^3+^ species in the *ex situ* Bi 4f XPS spectra (Figure 3c; Figure S13 and Table S1), confirm that metallic Bi and Bi_2_O_3_ domains do not fully vanish during deep redox cycles. Instead, they are continuously retained as residual heterophase nuclei. Additionally, the XRD pattern after 400 cycles maintains the biphasic features of the Bi/Bi_2_O_3_ HJ, verifying the structural robustness of these heterointerfaces during prolonged cycling (Figure S14).

This phase evolution indicates that the Bi/Bi_2_O_3_ heterostructure maintains a dynamically regulated Bi/Bi_2_O_3_ ratio throughout electrochemical cycling. Such balanced phase coexistence preserves abundant heterointerfaces while ensuring sufficient electronic conductivity, thereby promoting reversible phase conversion. Excessive Bi formation may weaken the heterointerfacial effect, whereas insufficient Bi generation may limit charge‐transfer kinetics. Notably, no diffraction features assignable to crystalline Bi_2_O_2_ were resolved by *ex situ* XRD, suggesting that any low‐valence Bi species, if formed, are likely transient and/or insufficiently ordered for XRD detection. Notably, no diffraction features assignable to crystalline Bi_2_O_2_ were resolved by *ex situ* XRD, suggesting that any low‐valence Bi species, if formed, are likely transient and/or insufficiently ordered for XRD detection [20].

To explicitly verify whether these continuously retained domains are responsible for the high‐rate performance, a targeted elimination experiment was performed. After potentiostatic oxidation of the Bi/Bi_2_O_3_ electrode at approximately −0.55 V vs. Hg/HgO, the current gradually decayed to nearly 0 mA, suggesting that the oxidation current had largely diminished under the applied potential (Figure 3d). Subsequent *ex situ* XRD results confirmed the complete disappearance of the Bi diffraction peaks, indicating that the crystalline Bi phase was reduced below the XRD detection limit, and that the initially detectable Bi/Bi_2_O_3_ phase coexistence was largely removed (Figure 3e). When the oxidized electrode was re‐evaluated via GCD at 50 A g^−1^, it exhibited a sharp potential drop and delivered negligible capacity (Figure 3f). This result strongly supports that the Bi/Bi_2_O_3_ heterointerface plays a critical role in enabling rapid Bi^0^/Bi^3+^ conversion at high rates, likely by providing pre‐existing nucleation‐active regions for reversible phase transformation.

The fundamental origin of this kinetic enhancement is comprehensively elucidated by *operando* electrochemistry and density functional theory (DFT) calculations. *Operando* electrochemical impedance spectroscopy (EIS) coupled with distribution of relaxation times (DRT) analysis corroborates the macroscopic kinetic advantages: Compared to Bi_2_O_3_, the Bi/Bi_2_O_3_ HJ exhibits attenuated and more concentrated relaxation signals in the charge‐transfer response (CT‐resp.) and the diffusion/phase‐transformation polarization response (DP‐resp.) regions (Figure 3g and Figure S15). These features indicate reduced interfacial charge‐transfer resistance and mitigated diffusion hindrance and phase‐transformation polarization of the Bi/Bi_2_O_3_ HJ during deep conversion. At the atomic and thermodynamic levels, DFT formation energy calculations (Figure 3h) reveal that Bi formation at the Bi/Bi_2_O_3_ heterointerface is thermodynamically more favorable than that on the pure Bi_2_O_3_ surface, with a lower formation energy (−3.102 vs. −1.190 eV). This profound disparity confirms that bypassing the energy‐demanding homogeneous nucleation step via direct interfacial regrowth is thermodynamically critical. Furthermore, charge density difference calculations show pronounced electron redistribution at the Bi/Bi_2_O_3_ interface, suggesting strong interfacial electronic coupling between metallic Bi and Bi_2_O_3_ (Figure 3i) [45].

The above findings systematically establish that the robust energy storage of the Bi/Bi_2_O_3_ HJ originates from a pre‐seeded, heterophase nuclei‐mediated conversion mechanism (Figure 3j). Following deep reduction, residual Bi_2_O_3_ domains can facilitate subsequent oxidation by serving as growth sites for Bi_2_O_3_. Conversely, following deep oxidation, residual metallic Bi domains can facilitate subsequent reduction by serving as nucleation‐active sites for Bi regrowth. Therefore, the bidirectional Bi^0^/Bi^3+^ conversion preferentially proceeds via heterophase regrowth along the pre‐existing Bi/Bi_2_O_3_ interfaces rather than relying on repeated, energy‐demanding homogeneous renucleation. This pathway systematically lowers the apparent nucleation overpotential, mitigates phase‐transition polarization, and effectively decouples the high‐rate reaction kinetics from the intrinsic limitations of the nucleation step, endowing the material with ultrafast and stable electrochemical reversibility [30, 46, 47].

### 2.4 N‐Doped Carbon‐Confined Bi/Bi_2_O_3_ Heterojunction for Fast and Stable Conversion

Building upon the Bi/Bi_2_O_3_ HJ core, this study further introduces a PDA‐derived N‐doped carbon (N—C) shell to construct a Bi/Bi_2_O_3_@C core–shell architecture. This design strategy aims to improve electronic transport and help maintain structural integrity during high rate cycling [18, 24, 25, 27].

Comparative electrochemical analyses were conducted to evaluate the effects of the Bi/Bi_2_O_3_@C core–shell structure. Furthermore, the influence of carbon confinement on the practical operating potential was evaluated by comparing the GCD profiles of pristine Bi_2_O_3_, Bi/Bi_2_O_3_, and Bi/Bi_2_O_3_@C electrodes (Figure S16). The apparent reduction plateau gradually shifts toward a more negative potential, confirming that the interfacial and conductive engineering strategies can effectively regulate the operating potential of Bi‐based electrodes [18, 48, 49]. The GCD curves of Bi/Bi_2_O_3_@C exhibit reduced apparent nucleation overpotentials and voltage (IR) drops across vario us current densities compared to the uncoated Bi/Bi_2_O_3_ electrode (Figures S17 and S18). Specifically, during the oxidation process at 1 A g^−1^, the IR drop is reduced from 0.5458 to 0.5236 V, while the apparent nucleation overpotential decreases from 0.0726 to 0.0659 V (Figure 4a). The reduced IR drop is consistent with lower overall ohmic polarization, likely associated with improved electronic connectivity in the carbon‐coated electrode. Concurrently, the lower apparent nucleation overpotential indicates that the N—C layer preserves the interfacial accessibility of pre‐seeded heterophase nuclei, enabling the Bi^0^/Bi^3+^ conversion to proceed efficiently via heterophase regrowth. Accordingly, the Nyquist plot (Figure S19) demonstrates that Bi/Bi_2_O_3_@C possesses lower charge‐transfer resistance and a mitigated Warburg diffusion impedance, indicating faster and more reversible Bi^0^/Bi^3+^ phase‐transition kinetics [18].

**FIGURE 4:**
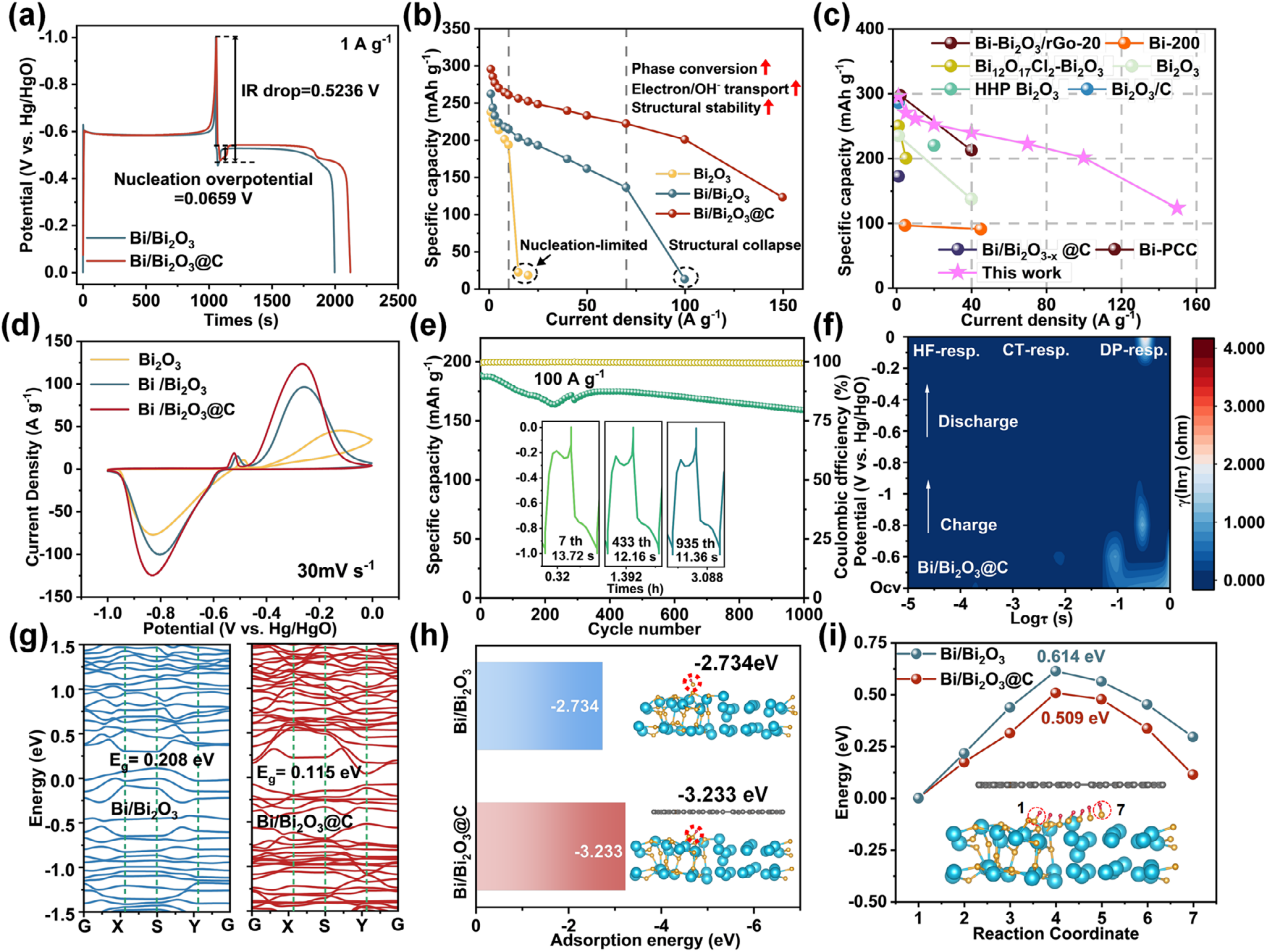
Electrochemical performance and mechanism of Bi/Bi_2_O_3_ and Bi/Bi_2_O_3_@C electrodes. (a) GCD curves at 1 A g^−1^. (b) Rate capability comparison and kinetic analysis. (c) Comparison with previously reported Bi‐based electrodes. (d) CV curves at 30 mV s^−1^. (e) Cycling stability at 100 A g^−1^ of Bi/Bi_2_O_3_@C. (f) The DRT plots of the Bi/Bi_2_O_3_@C electrode during the first charge–discharge cycle. (g) Calculated band structures (h) Calculated OH^−^ adsorption energies. (i) Calculated interfacial reaction energy profiles.

The Bi/Bi_2_O_3_@C electrode delivers high specific capacity and rate capability over a wide current‐density range of 1–150 A g^−1^, which is associated with accelerated Bi^0^/Bi^3+^ conversion at the internal heterointerfaces and enhanced electronic transport and structural stability provided by the outer N‐doped carbon layer (Figure 4b and Figures S20 and S21). At 1 A g^−1^, Bi/Bi_2_O_3_@C delivers a discharge specific capacity of 295.7 mAh g^−1^. Remarkably, even at ultrahigh current densities of 100 and 150 A g^−1^, presenting capacities of 201.07 and 123.1 mAh g^−1^, respectively. To further clarify the charge‐storage origin at high rates, the *b*‐value for the CV oxidation and reduction peaks are calculated to be 0.5070 and 0.5281, respectively, indicating that the redox process still retains a strong diffusion‐influenced conversion feature (Figure S22). As illustrated in Figure 4c and detailed in Table S3, Bi/Bi_2_O_3_@C shows much superior rate performance than state‐of‐the‐art Bi‐based aqueous anode materials [17, 19, 20, 50]. Furthermore, at a scan rate of 30 mV^−1^, Bi/Bi_2_O_3_@C displays the largest integrated area and the sharpest redox response among the evaluated samples, consistent with enhanced charge‐storage kinetics (Figure 4d and Figure S23). Notably, the enhanced capacity of Bi/Bi_2_O_3_@C does not originate from the intrinsic capacity contribution of the carbon shell, but rather from the improved utilization efficiency of the Bi/Bi_2_O_3_ active domains. The conformal N—C layer establishes continuous electron‐transport pathways around the heterojunction particles and strengthens interfacial electronic coupling, thereby reducing charge‐transfer limitations and enabling more electrochemically accessible Bi/Bi_2_O_3_ cregions to participate in the reversible Bi^0^/Bi^3+^ conversion [51].

Cycling and rate‐recovery tests further demonstrate that the outer N—C shell contributes to maintaining the electrochemical stability of the internal Bi/Bi_2_O_3_ heterojunction. At a current density of 1 A g^−1^, Bi/Bi_2_O_3_@C shows improved cycling stability, with a capacity retention of 55.45% after 300 cycles, compared with 45.75% for Bi/Bi_2_O_3_ and 20.65% for pristine Bi_2_O_3_ (Figure S24a). After 1000 cycles at current densities of 10, 50, and 100 A g^−1^, the Bi/Bi_2_O_3_@C electrode retains 73.8%, 82.0%, and 82.4% of its initial capacity, respectively (Figure 4e and Figure S24b). In the rate‐recovery test, when the current density is increased from 1 to 100 A g^−1^, Bi/Bi_2_O_3_@C robustly recovers its initial capacity, whereas the bare Bi/Bi_2_O_3_ electrode exhibits pronounced irreversible capacity loss (Figure S24c). Post‐cycling SEM analysis reveals that while the uncoated Bi/Bi_2_O_3_ undergoes severe structural collapse, Bi/Bi_2_O_3_@C maintains a relatively intact porous architecture (Figure S25). This morphological preservation indicates that the N—‐C layer effectively buffers phase transformation induced structural strain, suppressing active‐material aggregation and interfacial deactivation [29]. Correspondingly, *operando* DRT analysis of Bi/Bi_2_O_3_@C displays attenuated relaxation responses in both the CT‐resp. and DP‐resp. regions compared to Bi/Bi_2_O_3_ sample (Figure 4f). This reduction in interfacial charge‐transfer resistance and diffusion/ phase transformation polarization aligns seamlessly with the macroscopically observed lower IR drops and reduced apparent nucleation overpotentials. Undoubtedly, the construction of Bi/Bi_2_O_3_ heterointerfaces and the introduction of an N—C shell improve the conversion kinetics and cycling stability of the electrode. Self‐discharge behavior remains another important factor affecting the practical application of aqueous battery systems [52, 53]. Therefore, the self‐discharge characteristics of Bi/Bi_2_O_3_ and Bi/Bi_2_O_3_@C electrodes were evaluated after a 12 h resting period (Figure S26). As shown in Figure S26, the capacity retentions are calculated to be 89.80% and 90.84% for Bi/Bi_2_O_3_ and Bi/Bi_2_O_3_@C, respectively. The Bi/Bi_2_O_3_@C electrode exhibits slightly enhanced charge retention, which is likely related to the protective confinement effect of the N—C shell, helping preserve the structural integrity of the Bi/Bi_2_O_3_ heterointerface and mitigate undesired interfacial degradation during the resting period.

DFT calculations further support these electrochemical observations at the atomic level. Band structure analysis reveals that the integration of the N—C shell narrows the bandgap from 0.208 eV (for Bi/Bi_2_O_3_) to 0.115 eV (for Bi/Bi_2_O_3_@C), suggesting facilitated electron transport (Figure 4g and Figures S27 and S28) [54]. Projected density of states (PDOS) and charge density difference analyses further demonstrate that Bi/Bi_2_O_3_@C possesses a more continuous distribution of electronic states across the Fermi level alongside pronounced charge redistribution at the N—C/Bi/Bi_2_O_3_ boundaries (Figures S29 and S30). These features indicate enhanced interfacial electronic coupling [26, 55]. Furthermore, the OH^−^ adsorption energy on Bi/Bi_2_O_3_@C shifts from −2.734 to −3.233 eV, and the interfacial reaction barrier decreases from 0.614 to 0.509 eV (Figure 4h,i and Figure S31). These thermodynamic and kinetic modulations establish that the N—C layer optimizes intermediate OH^−^ adsorption and accelerates the overall interfacial reaction process [25].

Collectively, the outstanding rate performance of the Bi/Bi_2_O_3_@C arises from the synergistic effects of the internal Bi/Bi_2_O_3_ heterointerfaces and the outer N—C shell. The abundant internal Bi/Bi_2_O_3_ heterointerfaces serve as pre‐existing seed planes, enabling Bi^0^/Bi^3+^ conversion to proceed preferentially through interfacial regrowth rather than repeated nucleation. Concurrently, the outer N—C shell establishes continuous electron/OH^−^ transport networks and provides continuous electron‐transport pathways. This dual‐function design ensures that the core kinetic advantages of the heterophase conversion are sustained even under ultrahigh current densities.

### 2.5 Full‐Cell Demonstration

High‐mass‐loading electrodes in aqueous energy storage are typically hindered by prolonged electron‐transport pathways, restricted ion diffusion, and nonuniform reaction kinetics. While conventional studies often evaluate materials at idealized low mass loadings (1 – 2 mg cm^−2^), achieving stable electrochemical performance at practically relevant loadings (typically >10 mg cm^−2^) remains a critical threshold for commercial viability. To rigorously evaluate the Bi/Bi_2_O_3_@C architecture against this standard, thick electrodes with mass loadings progressively scaled from 2.4 to 12.4 mg cm^−2^ were tested. Notably, despite the substantial increase in electrode mass loading, the specific capacity does not suffer from the typical severe degradation. Constrained only by the maximum threshold of the testing instrument, the high‐loading electrode delivers robust and stable capacity output across a wide current density range of 1 to 25 A g^−1^ (Figure 5a and Figure S32) [24]. Specifically, the highest‐mass‐loading electrode (mass loading: 12.4 mg cm^−2^) exhibits a capacity retention of 96.2% from 1 to 25 A g^−1^ and retains 81.0% of its initial capacity after 1000 cycles at 10 A g^−1^ (Figure 5b). These results confirm that the conductive N—C network and the internal pre‐seeded Bi/Bi_2_O_3_ heterointerfaces synergistically sustain efficient charge transport and structurally stable reaction interfaces. This architectural design effectively overcomes the kinetic sluggishness and inner‐electrode passivation inherently associated with thick configurations, physically supporting rapid and reversible Bi^0^/Bi^3+^ conversion even under commercial‐level loading conditions [56].

**FIGURE 5:**
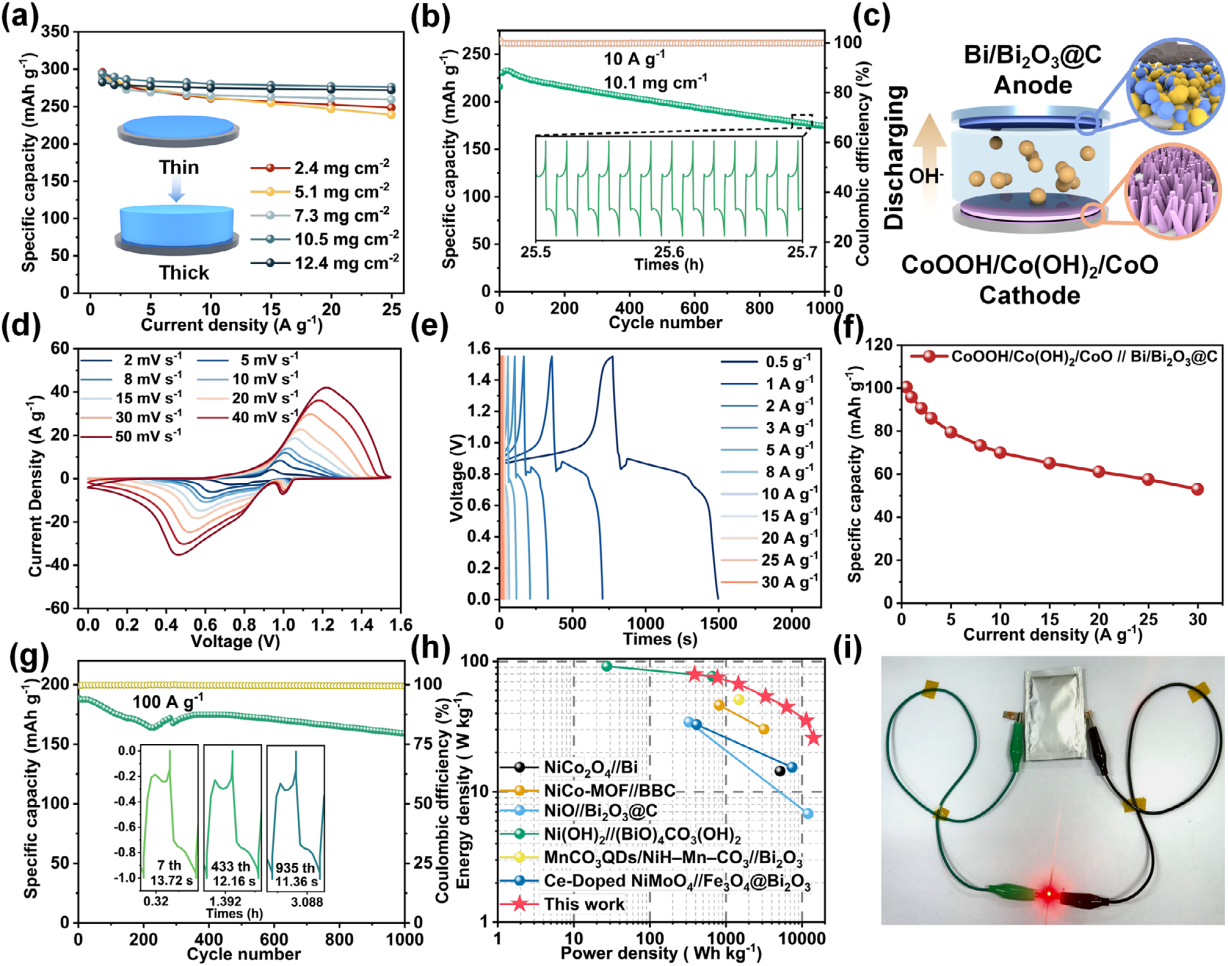
(a) Rate performance of Bi/Bi_2_O_3_@C electrodes with different mass loadings. (b) Cycling performance of the high‐mass‐loading electrode at 10 A g^−1^. (c) Schematic illustration of the CoOOH/Co(OH)_2_/CoO//Bi/Bi_2_O_3_@C assembled AABs. (d) CV curves at different scan rates. (e) GCD curves at various current densities. (f) Rate capability of the full cell. (g) Cycling stability of the full cell at 10 A g^−1^. (h) Ragone plot comparing the energy and power densities of the fabricated full cell with those of previously reported aqueous energy‐storage devices. (i) Photograph of a pouch cell device powering a light‐emitting diode.

To evaluate the full‐cell performance of the Bi/Bi_2_O_3_@C anode, CoOOH/Co(OH)_2_/CoO//Bi/Bi_2_O_3_@C AABs were assembled utilizing the Bi/Bi_2_O_3_@C anode and a self‐supported CoOOH/Co(OH)_2_/CoO nanoarrays cathode (Figures S33 and S34 and Figure 5c, Equations S7–S9). The complementary potential windows of the two electrodes enable a stable operating voltage window of approximately 1.55 V (Figures S35). The full cell exhibits distinct redox responses at different scan rates (Figure 5d) and well‐defined charge–discharge plateaus at different current densities, confirming reversible electrochemical reaction kinetics and appropriate mass/kinetic matching between the cathode and anode (Figure 5e). Moreover, the Nyquist plot of the AABs device reveals a low series resistance of 1.01 Ω, indicating favorable internal charge transport within the full cell (Figure S36). Based on the combined active‐material masses of the cathode and anode the full cell delivers a specific capacity of 100.4 mAh g^−1^ at 0.5 A g^−1^ and retains 52.92 mAh g^−1^ at 30 A g^−1^ (Figure 5f). During cycling at 10 A g^−1^, the full cell preserves 74.0% of its capacity after 1000 cycles, demonstrating stable operation under fast charge/discharge conditions (Figure 5g). Based on the active material mass of both cathode and anode, the assembled AABs yields an energy density of 79.6 Wh kg^−1^ at a power density of 390 W kg^−1^, and sustains 25.75 Wh kg^−1^ even at a high‐power density of 14,380 W kg^−1^ (Equations S4 and S5). As illustrated in the Ragone plot (Figure 5h) and summarized in Table S4, these energy‐power metrics compare favorably with recently reported bismuth based aqueous energy storage devices [17, 27, 50]. Additionally, a prototype CoOOH/Co(OH)_2_/CoO//Bi/Bi_2_O_3_@C pouch cell successfully powers an light‐emitting diode (LED) indicator, validating the practical feasibility of this system (Figure 5i).

While full‐cell performance is inherently governed by multiple systemic factors—including capacity matching, kinetic compatibility, mass‐loading ratios, and device configuration—the robust capacity output of the Bi/Bi_2_O_3_@C anode under high‐loading and full‐cell conditions highlights the effectiveness of its structural design. The synergistic combination of pre‐seeded heterophase nucleation sites and the N—C confining network effectively sustain high‐rate anode reactions, providing a feasible interfacial engineering strategy for high‐power aqueous batteries.

## 3 Conclusion

This study establishes a heterointerface‐seeded reversible conversion mechanism to reduce the intrinsic kinetic limitations of Bi‐based anodes. By structurally engineering Bi/Bi_2_O_3_@C core–shell nanowires, we demonstrate that continuously retained Bi and Bi_2_O_3_ domains act as mutually reversible nucleation sites during deep redox cycling. These heterointerfaces guide the bidirectional Bi^0^/Bi^3+^ conversion to proceed preferentially via interfacial regrowth, effectively reducing the reliance on the energy intensive homogeneous nucleation step and substantially reducing phase‐transition polarization. Concurrently, the outer N‐doped carbon shell maintains continuous electron/OH^−^ transport networks and provides robust structural confinement, preserving these interfacial kinetic advantages under high‐rate and high‐loading regimes. Consequently, the optimized Bi/Bi_2_O_3_@C electrode delivers 295.7 mAh g^−1^ at 1 A g^−1^ and retains 123.1 mAh g^−1^ at 150 A g^−1^, achieving a capacity retention of 82.4% after 1000 cycles at 100 A g^−1^. This structural stability persists under a high mass loading of 12.4 mg cm^−2^ (high capacity retention of 96.2% from 1 to 25 A g^−1^ and 81.0% retention after 1000 cycles at 10 A g^−1^). Furthermore, full‐cell AABs paired with a CoOOH/Co(OH)_2_/CoO cathode and the Bi/Bi_2_O_3_@C anode operate stably within a 1.55 V window, validating the practical viability of this material in prototype devices. Overall, this pre‐seeded heterojunction design, by decoupling conversion kinetics from nucleation barriers, provides a rational interfacial engineering strategy for fast‐charging aqueous batteries, while its generalizability to other nucleation‐limited multivalent metal anodes provides a distinct direction for future exploration.

## Author Contributions

**Jingwen Ma**: investigation, validation, visualization, Writing – original draft, formal analysis. **Teng Wang**: conceptualization, methodology, funding acquisition, project administration, resources, writing – review and editing. **Zhongguo Zhao**: methodology, software, validation, writing – review and editing. **Yuanyou Peng**: data curation, validation, visualization, writing – review and editing. **Meimei Yu**: writing – review and editing, resources, data curation, formal analysis. **Chaojiang Fan**: writing – review and editing, data curation, validation, formal analysis. **Wenhu Li**: methodology, supervision, project administration, writing – review and editing. **Jiaye Ye**: methodology, data curation, formal analysis, writing – review and editing. **Tong Yang**: writing – review and editing, visualization, data curation, formal analysis. **Hongxia Wang**: conceptualization, methodology, supervision, project administration, writing – review and editing.

## Conflicts of Interest

The authors declare that they have no known competing financial interests or personal relationships that could have appeared to influence the work reported in this paper.

## Supporting information




**Supporting File**: advs78066‐sup‐0001‐SuppMat.docx.

## Data Availability

The data that support the findings of this study are available from the corresponding author upon reasonable request.
