# Supplementary material for: Heterointerface Seeding Accelerates Bi0/Bi3+ Phase Conversion for Ultrafast Aqueous Alkaline Batteries
Source: Adv Sci (Weinh). 2026 Sep 30:e78066. Online ahead of print. doi: 10.1002/advs.78066 (PMC13626029; doi:10.1002/advs.78066)
Supplement: Supplementary file 1 [file ADVS-9999-e78066-s001.docx]

**Supporting information**

**Heterointerface Seeding Accelerates Bi^0^/Bi^3+^** **Phase Conversion for Ultrafast Aqueous Alkaline Batteries**

*Jingwen Ma, Teng Wang*,* *Jiaye Ye, Tong Yang, Zhongguo Zhao, Yuanyou Peng, Meimei Yu, Chaojiang Fan, Wenhu Li*, and Hongxia Wang**

Jingwen Ma, Teng Wang, Zhongguo Zhao, Yuanyou Peng, Meimei Yu, Chaojiang Fan, Wenhu Li

School of Materials Science and Engineering, Shaanxi University of Technology, Hanzhong 723001, Shaanxi, China.

Email: [wangtengjun_@126.com;](mailto:wangtengjun_@126.com;) ccwhli@163.com.

Jiaye Ye, Tong Yang, Hongxia Wang

School of Chemistry and Physics, Faculty of Science, Queensland University of Technology, Brisbane, Queensland 4001, Australia.

Email: [hx.wang@qut.edu.au](mailto:hx.wang@qut.edu.au).

* Corresponding authors.

**Experimental Section**

**Materials:** All the reagents and solvents were commercially available and used as supplied without further purification. Bismuth nitrate pentahydrate (Bi(NO_3_)_3_·5H_2_O), terephthalic acid (H_2_BDC), N,N-dimethylformamide (DMF), dopamine hydrochloride, Tris(hydroxymethyl)aminomethane hydrochloride (Tris-HCl), and potassium hydroxide (KOH) were all purchased from Shanghai Aladdin Biochemical Technology Co., Ltd. All chemicals and reagents were of analytical grade and used as received without further purification. Deionized (DI) water was used throughout the experiments.

**Preparation of the Bi_2_O_3_**

The Bi_2_O_3_ control sample was prepared according to a previously reported method with slight modifications**^[1]^**.

**Preparation of the Bi-MOF precursors**

In a typical synthesis, bismuth nitrate pentahydrate (Bi(NO_3_)_3_·5H_2_O) was dissolved in 18 mL of N,N-dimethylformamide (DMF) to form a 0.03 M solution. The mixture was magnetically stirred for 5 min and subsequently sonicated for 20 min to ensure complete dissolution. Then, terephthalic acid (H_2_BDC) was added to the above solution to achieve a concentration of 0.03 M. The resulting mixture was subjected to another round of stirring for 5 min and ultrasonication for 20 min to obtain a homogeneous solution. The solution was then transferred into a Teflon-lined stainless-steel autoclave and maintained at 120 ℃ for 24 h. After the reaction system cooled naturally to room temperature, the precipitate was collected and washed with anhydrous ethanol three times to remove unreacted residues. Finally, the obtained Bi-MOF precursors were dried in an oven at 60 ℃ for 12 h.

**Preparation of the Bi/Bi_2_O_3_ heterojunction nanowires**

The as-prepared dried Bi-MOF precursors were first ground into fine powders into a crucible. The powders were then subjected to thermal treatment in a muffle furnace under an air atmosphere. The calcination process was conducted at 350 ℃ for 2 h, with a heating rate of 5 ℃ min ^−1^. Finally, the furnace was allowed to cool naturally to room temperature to yield the final Bi/Bi_2_O_3_ heterojunction nanowires.

**Preparation of the Bi/Bi_2_O_3_@C Core-Shell Nanowires**

To construct the core-shell architecture, 200 mg of the as-prepared Bi/Bi_2_O_3_ composites were dispersed into 100 mL of Tris-HCl buffer solution (10 mM). The pH value of the suspension was adjusted to 8.5 to trigger the self-polymerization of dopamine. Subsequently, 200 mg of dopamine hydrochloride was added. The mixture was stirred for 30 min and then aged for 22 h at room temperature to form a uniform polydopamine (PDA) coating layer. The resulting intermediates (Bi/Bi_2_O_3_@PDA) were collected, washed with anhydrous ethanol three times, and dried at 60 ℃ for 12 h. Finally, the dried powders were placed in a porcelain boat and carbonized in a tube furnace at 600 ℃ for 2 h under an argon atmosphere with a heating rate of 2 ℃ min^−1^. After cooling naturally to room temperature, the Bi/Bi_2_O_3_@C core-shell nanowires were obtained.

**Preparation of the CoOOH/Co(OH)_2_/CoO**

Briefly, a piece of pre-cleaned nickel foam (NF,1×4 cm^2^) was plasma-treated to enhance hydrophilicity and partially masked with temperature-resistant tape. It was then immersed in a 17.0 mL aqueous solution containing 0.03 M Co(NO_3_)_2_∙6H_2_O, 0.09 M NH_4_F, and 0.15 M urea. After a hydrothermal reaction at 120 ℃ for 8 h, the as-grown Co(OH)_2_ nanowire arrays were collected, washed, and dried. Subsequently, the precursor was calcined in a Ar_2_ atmosphere at 400 ℃ for 2 h (heating rate of 5 ℃ min^−1^) to yield the Co(OH)_2_/CoO intermediate. Finally, the annealed Co(OH)_2_/CoO electrode underwent an in-situ ECA process by executing 45 cyclic voltammetry (CV) cycles in a voltage window of -1.0 to 0.65 V (vs. Hg/HgO) at 20 mV s^−1^ to derive the ultimately activated hierarchical arrays CoOOH/Co(OH)_2_/CoO.

**Characterization**

The crystal structure, molecular structure, and elemental composition of the samples were analyzed through X-ray diffractometer (XRD, Rigaku Mini Flex 600 with Cu Kɑ radiation), Raman spectra were recorded using a Raman spectrometer (WITec Alpha 300R), and X-ray photoelectron spectroscopy (XPS, ESCALAB Xi+) spectra corrected by C 1s line at 284.8 eV. The scanning electron microscope (SEM, FEI Apreo S HiVoc) and transmission electron microscope (TEM, Thermo Fisher-K-Alpha) were utilized to reveal the morphology evolution of the samples.

**Electrochemical Measurements**

The electrochemical properties of the active materials were tested by a three-electrode system and aqueous alkaline battery device(AABs). All electrochemical measurements were carried out with electrochemical workstation VSP, BioLogi and CHI 660E in 6 M KOH inner electrolyte at room temperature (25 ºC).

The anode was fabricated by a traditional slurry coating method. In brief, the anode material, acetylene black and polytetrafluoroethylene binder (5 wt%) with mass ratio of 8:1:1 was first mixed and ground in an agate mortar for 30 min before adding appropriate N-methyl pyrrolidinone (NMP) solvent to obtain a slurry, which was then coated on a piece of pre-cleaned nickel foam (1 × 1 cm^2^). After being dried in a vacuum oven at 60 ℃ for 8 h, the active materials with the other chemicals were pressed onto nickel foam at 5 MPa for 30 s in order to enhance the adhesion between the active materials and the current collector. The mass loadings of active material were varied from 2.0 to 13.0 mg cm^-2^.

The three-electrode test system includes a working electrode, a HgO/Hg reference electrode (in 1 M KOH inner electrolyte) and a Pt foil auxiliary electrode immersed in 6 M KOH electrolyte. All of the potentials in this work were recorded relative to HgO/Hg reference electrode unless otherwise noted. The measurement includes cyclic voltammetry (CV), Galvanostatic charging-discharging (GCD), and electrochemical impedance spectroscopy (EIS) tested. The CV curves were carried out in different scan rates and the GCD and Cycling performance was tested at various current densities from 1 to 150 A g^−1^ in the potential window of -1 to 0 V. The EIS was tested at different frequencies from 0.01 Hz to 20.0 kHz under open circuit voltage with an alternate current amplitude of 5.0 mV. The accuracy of the electronic balance is d =0.01 mg (METTLER TOLEDO-ME55). The *Cs* (mAh g^−1^) of the electrode was calculated based on the discharge time by Equation (1)**^[2]^**:

$C_{s}=\frac{I\times\Delta t}{m}$	(1)

Where C_s_ (mAh g^−1^) is the specific capacity, I (mA) is the discharging current, Δt (h) is the discharging time and m (g) is the mass of active materials of working electrode.

For AABs, CoOOH/Co(OH)_2_/CoO as the cathode, Bi/Bi_2_O_3_@C anode. The as-prepared binder-free CoOOH/Co(OH)_2_/CoO was directly used as the cathode. Therefore, the mass of CoOOH/Co(OH)_2_/CoO needs to recalculation according to the charge balance principles. The positive and negative charge balance principles were used to assemble the asymmetric device according to Equation (2):

$\frac{m_{+}}{m_{-}}=\frac{C_{-}}{C_{+}}$	(2)

Where C_-_ and C_+_ mean the specific capacity of the anode and cathode (mAh g^−1^), m_+_ and m_-_ represent the mass of active materials (mg), respectively.The specific capacity C_cell_ (mAh g^−1^) of the AABs was also obtained through their corresponding GCD results based on Equation (3):

$C_{cell}=\frac{I\Delta t}{m}$	(3)

Where C_cell_ (mAh g^−1^) is the specific capacity, I (mA) is the discharging current, Δt (h) is the discharging time and and m (g) is the total mass of the active materials of the Bi/Bi_2_O_3_@C and CoOOH/Co(OH)_2_/CoO electrodes.

The energy density E and power density P of the AABs were obtained from the following Equations (4)(5)**^[3]^**:

$E=\frac{I\times\int_{0}^{\Delta t} Vdt}{3.6m}$	(4)

$P=\frac{3600E}{\Delta t}$	(5)

Where E is the energy density (Wh kg^−1^), P is the power density (W kg^−1^), I is the discharge current (A), V is the discharging voltage (V), Δt is the discharging time (s), dt is the time differential, m is the total mass of the active materials of the Bi/Bi_2_O_3_@C and CoOOH/Co(OH)_2_/CoO electrodes (g).

**Details of density function theory (DFT) calculations**

All the calculations were performed within the framework of the density functional theory (DFT) as implemented in the Vienna Ab initio Software Package (VASP) code within the Perdew-Burke-Ernzerhof (PBE) generalized gradient approximation and the projected augmented wave (PAW) method. The cutoff energy for the plane-wave basis set was set to 520 eV**^[4]^**. Monkhorst-Pack special k-point meshes of 3 × 3 × 1 were proposed to carry out geometry optimization and electronic structure calculation**^[5]^**. During the geometry optimization, all atoms were allowed to relax without any constraints until the convergence thresholds of maximum force and energy were smaller than 0.01 eV/Å and 1.0 × 10^−5^ eV/atom, respectively. A vacuum layer of 15 Å was introduced to avoid interactions between periodic images. The intermolecular van der Waals forces were corrected using the method of DFT-D3**^[6]^**. The adsorption energy (E_ads_) of the anions is calculated by the equation: E_ads_ = E_total_ - E_catalyst_ - E_mol_, in which the E_total_ represents the total energy; E_catalyst_ represents the catalyst model energy; and E_mol_ represents the energy of adsorbed anions**^[7]^**.

The Gibbs free energy of each elementary step was calculated as Equation (6)：

ΔG =ΔE + ΔZPE -TΔS	(6)

Where ΔE is the total energy difference between the pristine surface and adsorbing surface calculated. ΔZPE and ΔS refer to the change in zero-point energies and entropy during the reaction, respectively. The Gibbs free energy correction for enthalpy and entropy at standard condition (1 atm, 298.15 K) was considered.

In the CoOOH/Co(OH)_2_/CoO//Bi/Bi_2_O_3_@C AABs, the energy storage process can be mainly described by the reversible Bi_2_O_3_/Bi conversion at the anode and the Co^2+^/Co^3+^ redox reaction at the cathode**^[8]^**. The reaction is described as Equation (7)-(9):

$\text{Bi}_{\text{2}}\text{O}_{\text{3}}\text{ + 3}\text{H}_{\text{2}}\text{O + 6}\text{e}^{\text{-}}\text{ }\text{↔ 2}\text{Bi}\text{ + 6}\text{OH}^{\text{-}}$	(7)

$\text{Co(OH}\text{)}_{\text{2}}\text{ + O}\text{H}^{\text{-}}\text{ ↔ CoOOH + }\text{H}_{\text{2}}\text{O + }\text{e}^{\text{-}}$	(8)

$\text{CoO + O}\text{H}^{\text{-}}\text{ ↔ CoOOH + }\text{e}^{\text{-}}$	(9)

The following Equation (10) allows for a more precise quantitative estimation of the contributions from capacitive behavior and the diffusion-controlled process:

$\text{i}\text{(}\text{V}\text{) = }\text{k}_{\text{1}}\text{v}\text{ + }\text{k}_{\text{2}}\text{v}^{\text{1/2}}$	(10)

Where *v* is scanning rate, k_1_ and k_2_ are adjustable values. The total current *i*(V) can be divided into two parts, one is related to surface capacitance (k_1_*v*) and another is related to diffusion control (k_2_*v*^1/2^).

**Figure S1.** SEM images of a) Bi-MOF nanorods (NRs), b) Bi/Bi_2_O_3_, c) Bi/Bi_2_O_3_@C and d) Bi_2_O_3_.

**Figure S2.** _._HRTEM image of the Bi/Bi_2_O_3_ heterostructure. The enlarged lattice-fringe images reveal the characteristic lattice spacings of Bi, corresponding to the (012), (110), and (104) planes, and Bi_2_O_3_, corresponding to the (201), (311) and (220) planes, respectively. The coexistence of these distinct crystalline domains within the same nanowire and their intimate nanoscale contact further confirm the formation of the Bi/Bi_2_O_3_ heterointerfaces.

**Figure S3.** XPS spectra of Bi/Bi_2_O_3_ and Bi/Bi_2_O@C, a) Survey spectrum, b) O 1s c) C1s and d) N 1s.

**Figure S4.** GCD curves of Bi_2_O_3_.

**Figure S5.** Charge-discharge specific capacity of a) Bi_2_O_3_ and b) Bi/Bi_2_O_3_.

**Figure S6.** GCD curves of Bi_2_O_3_ and Bi/Bi_2_O_3_ at a) 10 A g^−1^ and b) 15 A g^−1^.

**Figure S7.** CV curves of Bi_2_O_3_ at 2-50 mV s^−1^.

**Figure S8.** Diffusion controlled and capacitive controlled contribution of a) Bi_2_O_3_ and b) Bi/Bi_2_O_3_ at different scan rates.

**Figure S9.** Comparison of IR drops for Bi_2_O_3_ and Bi/Bi_2_O_3_ at different current densities.

**Figure S10.** The XRD patterns and corresponding Rietveld refinement results of state 1.

**Figure S11.** *Ex-situ* TEM, HRTEM and EDS mapping of Bi/Bi_2_O_3_ electrodes at representative electrochemical states. a) State 4, b) State 6, c) State 7.

**Figure S12.** Ex-situ Raman spectra obtained during the discharging processes of Bi/Bi_2_O_3_.

**Figure S13.** Ex-situ O 1s XPS spectra of Bi/Bi_2_O_3_ at state 4 and state 7.

**Figure S14.** XRD pattern after 400 cycles of Bi/Bi_2_O_3_.

**Figure S15.** The DRT plots of Bi_2_O_3_ electrode during the first charge-discharge cycle.

**Figure S16.** GCD profiles of pristine Bi_2_O_3_, Bi/Bi_2_O_3_, and Bi/Bi_2_O_3_@C at 1 A g^−1^, highlighting the progressive shift of the apparent reduction plateau toward more negative potentials.

**Figure S17.** Comparison of apparent nucleation overpotential for Bi/Bi_2_O_3_ and Bi/Bi_2_O_3_@C at different current densities.

**Figure S18.** Comparison of IR drops for Bi/Bi_2_O_3_ and Bi/Bi_2_O_3_@C at different current densities.

**Figure S19.** Nyquist plots of Bi/Bi_2_O_3_ and Bi/Bi_2_O_3_@C.

**Figure S20.** GCD curves of Bi/Bi_2_O_3_@C at 1-150 A g^−1^.

**Figure S21.** Charge-discharge specific capacity of Bi/Bi_2_O_3_@C at 1-150 A g^−1^.

**Figure S22.** The relationship between the peak current and scan ratesof Bi/Bi_2_O_3_@C at 2-50 mV s^−1^.

**Figure S23.** CV curves of Bi/Bi_2_O_3_@C at 2-50 mV s^−1^.

**Figure S24.** a) Cycling performance of Bi_2_O_3_, Bi/Bi_2_O_3_ and Bi/Bi_2_O_3_@C at 1 A g^-1^. b) Cycling performance of Bi/Bi_2_O_3_@C at 10 A g^-1^ and 50 A g^-1^. c) Current-switching performance of Bi/Bi_2_O_3_ and Bi/Bi_2_O_3_@C.

**Figure S25.** SEM image of a) Bi/Bi_2_O_3_ and b) Bi/Bi_2_O_3_@C cycled 100 cycles at 100A g^−1^.

**Figure S26.** Self-discharge performance of the as-prepared electrodes. Charge-discharge specific capacity of a) Bi_2_O_3_, b) Bi/Bi_2_O_3_, and c) Bi/Bi_2_O_3_@C electrodes (the blue, yellow, brown and green curves represent complete discharge reaction, complete charge reaction, a resting process for 12 h and subsequent residual discharge reaction, respectively, all measured at a current density of 1 A g^−1^). d) Open-circuit potential evolution of the three electrodes during the 12 h resting period. e) Comparison of capacity retention ratios for the three electrodes after 12 h self-discharge.

**Figure S27.** Side view of structural models of Bi_2_O_3_ , Bi/Bi_2_O_3_ and Bi/Bi_2_O_3_@C.

**Figure S28.** Top view of structural models of Bi/Bi_2_O_3_@C.

**Figure S29.** Charge density difference of the Bi/Bi_2_O_3_@C heterointerface.

**Figure S30.** Calculated PDOS of Bi/Bi_2_O_3_ and Bi/Bi_2_O_3_@C.

**Figure S31.** Theoretical models along the interfacial reaction coordinate of Bi/Bi_2_O_3_.

**Figure S32.** GCD curves of the Bi/Bi_2_O_3_@C electrode at different active-mass loadings.

**Figure S33.** SEM images of CoOOH/Co(OH)_2_/CoO.

**Figure S34.** (a) CV curves, b) GCD curves and c) Rate performance of CoOOH/Co(OH)_2_/CoO.

**Figure S35.** CV curves of the individual cathode and anode electrodes at 20 mV s^−1^.

**Figure S36.** Nyquist plot of the AABs.

**Table S1.** Summarized XPS quantification data of the state 4 and state 7 of Bi/Bi_2_O_3_.

|  | **Samples** | **State 7** | **State 4** |
| --- | --- | --- | --- |
| **Wide** | C 1s | 66.67% | 67.49% |
|  | O 1s | 29.28% | 29.58% |
|  | Bi4f | 4.04% | 2.93% |
| **O 1s** | Bi-O | 8.35% | 5.32% |
|  | Bi-OH | 29.87% | 8.75% |
|  | O-C=O/Bi-O-C | 49.48% | 51.60% |
|  | Adsorbed O | 12.30% | 34.33% |
| **Bi 4f** | Bi-O | 95.47% | 79.56% |
|  | Bi-Bi | 4.53% | 20.44% |
| **C 1s** | C-C | 41.40% | 34.93% |
|  | C-O/C-N | 38.95% | 35.79% |
|  | C=O | 5.03% | 7.28% |
|  | O-C=O | 14.63% | 22.0% |

**Table S2.** Summarized XPS quantification data of the Bi/Bi_2_O_3_ and Bi/Bi_2_O_3_@C.

|  | **Samples** | **Bi/Bi_2_O_3_** | **Bi/Bi_2_O_3_@C** |
| --- | --- | --- | --- |
| **Wide** | C 1s | 36.1% | 79.13% |
|  | N 1s | 5.91% | 8.29% |
|  | O 1s | 39.26% | 10.95% |
|  | Bi 4f | 18.73% | 1.62% |
| **O 1s** | Bi-O | 21.43% | 30.17% |
|  | Bi-OH | 48.96% | 8.85% |
|  | O-C=O/Bi-O-C | 18.99% | 47.22% |
|  | Adsorbed O | 10.63% | 13.76% |
| **Bi 4f** | Bi-O | 98.30% | 96.70% |
|  | Bi-Bi | 1.7% | 3.3% |

**Table S3.** Summary of the storage performance of Bi/Bi_2_O_3_@C.

| **Materials** | **Electrolyte** | **Current density (A g^−1^)** | **Specific capacity (mAh g^−1^)** | **References** |
| --- | --- | --- | --- | --- |
| Bi-Bi_2_O_3_/rGO-20 | 6 M KOH | 1 | 288.0 | [9] |
| Bi-200 | 1 M KOH | 4.5 | 96.2 | [10] |
|  |  | 45 | 90.5 |  |
| Bi_12_O_17_Cl_2_-Bi_2_O_3_ | 1 M KOH | 1 | 250.0 | [11] |
|  |  | 5 | 200.0 |  |
| α/β-Bi_2_O_3_ | 2 M KOH | 1 | 235.0 | [12] |
|  |  | 40 | 137.0 |  |
| HHP Bi_2_O_3_ | 6 M KOH | 20 | 220.0 | [13] |
| Bi_2_O_3_/C | 2 M KOH | 1 | 285.8 | [14] |
| ACFP/α-Bi_2_O_3_ | 6 M KOH | 1 | 251.6 | [8] |
|  |  | 15 | 200.0 |  |
| h-Bi_2_O_3_ | 6 M KOH | 1 | 289.7 | [15] |
|  |  | 5 | 258.6 |  |
|  |  | 20 | 229.4 |  |
|  |  | 50 | 178.3 |  |
|  |  | 60 | 155.6 |  |
| Bi/Bi_2_O_3-x_@C | 1 M KOH | 1 | 172.2 | [2] |
| Bi@NPCF | 6 M KOH | 3 | 196.1 | [16] |
| δ-Bi_2_O_3_ | 1 M KOH | 0.2 | 264 | [17] |
|  |  | 2 | 150 |  |
| Bi_2_O_3_/N-HPC | 6 M KOH | 0.5 | 190.3 | [18] |
|  |  | 20 | 138.9 |  |
| Bi_2_O_3_-20 | 6 M KOH | 0.5 | 245.1 | [19] |
| Bi-PCC | 6 M KOH | 2 | 297.1 | [20] |
|  |  | 40 | 212.4 |  |
| Bi/Bi_2_O_3_@C | 6 M KOH | 1 | 295.7 | This work |
|  |  | 5 | 270.28 |  |
|  |  | 10 | 261.1 |  |
|  |  | 20 | 252.4 |  |
|  |  | 40 | 239.8 |  |
|  |  | 70 | 222.31 |  |
|  |  | 100 | 201.1 |  |
|  |  | 150 | 123.1 |  |

**Table S4.** Summary of the storage performance of Bi-based AABs.

| **Energy storage device** | **C_p_ (mAh g^−1^)** | **P (W kg^−1^)** | **E (Wh kg^−1^)** | **Reference** |
| --- | --- | --- | --- | --- |
| Bi-Bi_2_O_3_/rGO-20//Zn | 134.4 mAh g^−1^  (1 A g^-1^) | **10078.7** | 48.8 | [9] |
| Ni/Bi battery | 113.0 mAh g^−1^ (0.2 C) | 27.3 | 92 | [21] |
|  |  | 670 | 77 |  |
| NiCo-MOF//BBC | 36.1 mAh g^−1^  (1 A g^-1^) | 823.4 | 46.2 | [2] |
|  |  | 3198 | 30.2 |  |
| Bi_12_O_17_Cl_2_-Bi_2_O_3_//CoNiS_x_ | 29.0 mAh g^−1^  (10 A g^-1^) | 5200 | 14.4 | [11] |
| MnCO_3_QDs/NiH–Mn–CO_3_//B-Bi_2_O_3_ | 68.3 mAh g^−1^  (2.0 A g^-1^) | 1500 | 51 | [22] |
| Ce-doped NiMoO_4_//Fe_3_O_4_@Bi_2_O_3_ | 47.3 mAh g^−1^  (1.0 A g^-1^) | 415 | 32.8 | [23] |
|  |  | 7481.8 | 15.4 |  |
| CoOOH/Co(OH)_2_/CoO//Bi/Bi_2_O_3_@C | 100.4 mAh g^−1^  (0.5 A g^-1^)  70.0 mAh g^−1^  (10 A g^-1^)  52.9 mAh g^−1^  (30 A g^-1^) | 390 | 79.6 | This work |
|  |  | 3349 | 53.93 |  |
|  |  | 14380 | 25.75 |  |

**References**

1	T. L. Wang, C. Cheng, Z. F. Guan, T. Tao, Q. D. Xiao, J. L. Zhu, “Chemical reduction-induced defect-rich bismuth oxide-reduced graphene oxide anode for high-performance supercapacitors,” *Journal of Colloid and Interface Science* 677 (2025): 45-54, <https://doi.org/10.1016/j.jcis.2024.07.213>.

2	C. Q. Zhang, L. Zhang, G. Feng, S. Z. Zheng, Z. H. Lu, “Multiscale Bi/Bi_2_O_3_ Mott-Schottky heterojunction confined in hierarchical mesoporous carbon for advanced aqueous alkaline batteries,” *Journal of Colloid and Interface Science* 714 (2026), <https://doi.org/10.1016/j.jcis.2026.140233>.

3	X. D. Wang, F. M. Zhou, Q. Y. Liang, et al., “Approaching Theoretical Capacity: 0D/2D Amorphous/Crystalline Bi-Based Heterostructures Anode for Aqueous Alkaline Rechargeable Batteries,” *Advanced Functional Materials* 34 (2024): 2408203, <https://doi.org/10.1002/adfm.202408203>.

4	B. Long, X. Y. Ma, L. J. Chen, et al., “Se Vacancy Activated Bi_2_Se_3_ Nanodots Encapsulated in Porous Carbon Nanofibers for Aqueous Zinc and Ammonium Ion Batteries,” *Advanced Functional Materials* 34 (2024): 2411430, <https://doi.org/10.1002/adfm.202411430>.

5	H. J. Monkhorst, J. D. Pack, “Special points for Brillouin-zone integrations,” *Physical Review B* 13 (1976): 5188-5192, <https://doi.org/10.1103/PhysRevB.13.5188>.

6	S. Grimme, J. Antony, S. Ehrlich, H. Krieg, “A consistent and accurateab initioparametrization of density functional dispersion correction (DFT-D) for the 94 elements H-Pu,” *The Journal of Chemical Physics* 132 (2010): 154104, <https://doi.org/10.1063/1.3382344>.

7	J. K. Nørskov, F. Abild-Pedersen, F. Studt, T. Bligaard, “Density functional theory in surface chemistry and catalysis,” *Proceedings of the National Academy of Sciences* 108 (2011): 937-943, <https://doi.org/10.1073/pnas.1006652108>.

8	J. Xu, Z. S. Meng, Z. Y. Hao, et al., “Oxygen-vacancy abundant alpha bismuth oxide with enhanced cycle stability for high-energy hybrid supercapacitor electrodes,” *Journal of Colloid and Interface Science* 609 (2022): 878-889, <https://doi.org/10.1016/j.jcis.2021.11.081>.

9	M. M. Shi, M. S. Zhao, Q. Y. Zheng, et al., “Uniform Bi-Bi_2_O_3_ nanoparticles/reduced graphene oxide composites for high-performance aqueous alkaline batteries,” *Dalton Transactions* 51 (2022): 12114-12124, <https://doi.org/10.1039/d2dt01217k>.

10	Y. Zeng, Z. Lin, Y. Meng, et al., “Flexible Ultrafast Aqueous Rechargeable Ni//Bi Battery Based on Highly Durable Single‐Crystalline Bismuth Nanostructured Anode,” *Advanced Materials* 28 (2016): 9188-9195, <https://doi.org/10.1002/adma.201603304>.

11	J. L. Chang, T. N. Wang, J. H. Ren, D. P. Wu, K. Jiang, Z. Y. Gao, “Bismuth oxychloride-bismuth oxide hybrid as a high-capacity anode material for aqueous alkaline battery,” *Journal of Colloid and Interface Science* 681 (2025): 229-238, <https://doi.org/10.1016/j.jcis.2024.11.191>.

12	K. S. Yin, D. L. Zhou, K. Tan, et al., “A space-confined strategy towards stable α/β-Bi_2_O_3_ heterojunction anode for high-energy aqueous battery,” *Journal of Colloid and Interface Science* 651 (2023): 929-937, <https://doi.org/10.1016/j.jcis.2023.08.035>.

13	G. T. Zan, T. Wu, P. Hu, et al., “An approaching-theoretical-capacity anode material for aqueous battery: Hollow hexagonal prism Bi_2_O_3_ assembled by nanoparticles,” *Energy Storage Materials* 28 (2020): 82-90, <https://doi.org/10.1016/j.ensm.2020.02.027>.

14	X. Sun, S. Wu, L. H. Chen, et al., “The construction of high-performance asymmetric supercapacitors by Bi-MOF-derived carbon skeleton with in situ activated Bi_2_O_3_/C and B-rGO/ amorphous NiCoB,” *Chemical Engineering Journal* 522 (2025): 167546, <https://doi.org/10.1016/j.cej.2025.167546>.

15	S. J. Yang, L. B. Qian, Y. J. Ping, et al., “Electrochemical performance of Bi_2_O_3_ supercapacitors improved by surface vacancy defects,” *Ceramics International* 47 (2021): 8290-8299, <https://doi.org/10.1016/j.ceramint.2020.11.190>.

16	K. Zhou, S. Wang, X. Y. Guo, et al., “Bismuth Nanoparticles Encapsulated in Nitrogen-Rich Porous Carbon Nanofibers as a High-Performance Anode for Aqueous Alkaline Rechargeable Batteries,” *Small* 18 (2022): 2105770, <https://doi.org/10.1002/smll.202105770>.

17	T. Qin, X. Zhang, D. Wang, et al., “Oxygen Vacancies Boost δ-Bi_2_O_3_ as a High-Performance Electrode for Rechargeable Aqueous Batteries,” *ACS Applied Materials & Interfaces* 11 (2018): 2103-2111, <https://doi.org/10.1021/acsami.8b19575>.

18	W. W. Zhu, D. K. Shen, “Synthesis of Bi_2_O_3_/hierarchical porous carbon composites for supercapacitor application,” *Journal of Energy Storage* 79 (2024): 110118, <https://doi.org/10.1016/j.est.2023.110118>.

19	J. F. Li, D. D. Chen, Q. W. Zhang, et al., “Synthesis of sponge-like Bi_2_O_3_ via a soft/hard-combined biomembrane support system for supercapacitor application,” *European Journal of Inorganic Chemistry* 15 (2018): 1688-1692, <https://doi.org/10.1002/ejic.201800151>.

20	H. Q. Shi, C. D. Zhang, J. M. Zhan, et al., “Bi Nanosheets on Porous Carbon Cloth Composites for Ultrastable Flexible Nickel-Bismuth Batteries,” *ACS Applied Materials & Interfaces* 15 (2023): 36190-36200, <https://doi.org/10.1021/acsami.3c05666>.

21	J. Sun, J. Wang, Z. Li, L. Niu, W. Hong, S. Yang, “Assembly and electrochemical properties of novel alkaline rechargeable Ni/Bi battery using Ni(OH)_2_ and (BiO)_4_CO_3_(OH)_2_ microspheres as electrode materials,” *Journal of Power Sources* 274 (2015): 1070-1075, <https://doi.org/10.1016/j.jpowsour.2014.10.172>.

22	N. M. Shinde, Q. X. Xia, J. M. Yun, R. S. Mane, K. H. Kim, “Polycrystalline and Mesoporous 3-D Bi_2_O_3_ Nanostructured Negatrodes for High-Energy and Power-Asymmetric Supercapacitors: Superfast Room-Temperature Direct Wet Chemical Growth,” *ACS Applied Materials & Interfaces* 10 (2018): 11037-11047, <https://doi.org/10.1021/acsami.8b00260>.

23	S. Z. Cui, F. Q. Wang, K. J. Sun, et al., “High-Performance Hybrid Supercapacitors Based on Ce-Doped NiMoO_4_ Nanosheets and Fe_3_O_4_@Bi_2_O_3_ Nanoarrays,” *Journal of Physical Chemistry C* 125 (2021): 18129-18140, <https://doi.org/10.1021/acs.jpcc.1c05387>.
